# Isolation and Characterization of Bacteriophages Infecting Nocardioforms in Wastewater Treatment Plant

**DOI:** 10.1155/2014/151952

**Published:** 2014-07-22

**Authors:** Krishna Khairnar, Preeti Pal, Rajshree H. Chandekar, Waman N. Paunikar

**Affiliations:** ^1^Environmental Virology Cell, Council of Scientific and Industrial Research, National Environmental Engineering Research Institute (CSIR-NEERI), Nehru Marg, Nagpur, Maharashtra 440020, India; ^2^School of Environment and Earth Science, North Maharashtra University, Jalgaon, Maharashtra 425001, India

## Abstract

Activated sludge plants (ASP) are associated with the stable foaming problem worldwide. Apart from the physical and chemical treatment methods, biological treatment method has been least explored and may prove to be a novel and ecofriendly approach to tackle the problem of stable foam formation. In ASP *Nocardia* species are commonly found and are one of the major causes for forming sticky and stable foam. This study describes the isolation and characterization of three *Nocardia* bacteriophages NOC1, NOC2, and NOC3 for the control of *Nocardia* species. The bacteriophages isolated in this study have shown promising results in controlling foam producing bacterial growth under laboratory conditions, suggesting that it may prove useful in the field as an alternative biocontrol agent to reduce the foaming problem. To the best of our knowledge to date no work has been published from India related to biological approach for the control of foaming.

## 1. Introduction

Activated sludge process (ASP) is the most commonly used process to reduce the toxicity of waste water by processing it microbiologically. In activated sludge system, treatment is given to every type of wastewater using microbial communities for the degradation of organic matter present in water [1]. Microbes use the organic matters as their energy source and degrade them into a less toxic form, but most of the system suffers from the excessive growth of unwanted mycolic acid containing filamentous bacteria or mycolata [2], which lead to the formation of brown and sticky foam [3]. Mycolata like* Sphaerotilus *spp*., Leptothrix *spp*., Microthrix parvicella, Corynebacterium *spp*., Dietzia *spp*., Nostocoida limicola, Gordonia *spp*., Skermania *spp*., Mycobacterium *spp*., Nocardia *spp*., Rhodococcus *spp*., Tsukamurella *spp., Type 021N, and Type 0041 play a role in foaming. However, among all mycolata the Eikelboom species are mainly responsible for foaming [4–7].

The foam covers a large area in the aeration tank which create interference in the treatment process and ultimately leads to the major environmental, operational, cosmetic, and health problems [3, 8, 9]. The outer surface of foam forming bacteria has hydrophobic properties similar to that of fats, oils, and grease which enables the bacterial bulk to float on the surface of liquid in aeration tank. The wastewater containing slowly degradable organic material like lipids, proteins, and fats may favor the growth of filamentous microorganisms like* M*.* parvicella* and* G. amarae* thereby leading to increased foaming [3, 6, 10]. The presence of unfavorable conditions in the activated sludge system, such as toxic conditions (pH below 6.5 or above 9.0), insufficient dissolved oxygen (DO), nutrient deficiencies, or seasonal temperatures may contribute to the foaming. It has been reported that the formation of scum and stable foam in aeration tank (AT), secondary clarifier (SC), and activated sludge (AS) is a global problem [9, 11, 12].

Various studies suggest that a range of physical, chemical, and biological [11, 13–15] methods is available for the control of foaming in activated sludge process [12], but they need a higher cost of maintenance. Current approaches for controlling foam includes decreasing mean cell retention time [16], use of classifying selectors, and nonspecific measures such as water sprays, steam application [17], polymer addition [18], and chlorination [19]. Dosing with cationic polymer [18] and controlling dissolved oxygen levels in the preoxidation reactor have been reported as useful methods for foam reduction [20].

There are only limited reports related to biological foam control methods especially the application of bacteriophages in AS systems [11, 14, 15, 21–23]. Bacteriophage therapy for the treatment of infectious diseases has shown promise [24]; on the similar lines lytic bacteriophages has the potential to be exploited as an biocontrol for filamentous bacteria which may lead to foam reduction in the AS treatment plant [11, 25]. Efforts have been made by Thomas et al. [23] and Petrovski et al. [13–15] for the isolation of bacteriophages against filamentous bacteria responsible for the stable foam formation in ASP. The successful application of phage for effective bacterial control relies on the population density which must be sufficient to support phage replication [26].

Bacteriophage based approach may have the potential as an environmentally safe option for tackling worldwide ASP operational problem of foaming. Nevertheless, phage-based foam control approach has certain limitations: (1) high concentration of phages must be applied for the successful application; (2) due to the polyvalent phages, broader host range could lead to the degradation of useful bacteria; (3) specific phage must be identified by the operator to counter specifically the foam forming bacteria without affecting other bacteria; (4) the microbial analysis of the system is a prerequisite to phage application as the bacterial population may vary between wastewater treatment plants (WWTP) [11].

The present research has been done with the objective of developing a biocontrol approach to manage foaming within AS systems in Nagpur city of India. In this study, we report the characterization of three* Nocardia* phages NOC1, NOC2, and NOC3 isolated from effluent treatment plant (ETP) and dairy ETP Nagpur, India. To the best of our knowledge to date, no work has been published from India related to biological approach for the control of foaming in WWTP.

## 2. Materials and Method

### 2.1. Sampling Sites

The sampling was done from effluent treatment plant (ETP) and dairy ETP around Nagpur, India, considering the foaming status of the treatment plants. As filamentous bacteria, for example,* Nocardia,* grows well on the slowly degradable organic matters like lipids, oil, and grease, fatty matters [3, 5, 10] and is found almost in every WWTP. So, in expectation of getting phages against* Nocardia* spp. the samples were collected from the above mentioned sites. Samples were collected from different points, namely, AT, SC, and AS, in sterile plastic bottles of 200 mL and processed within two days of sampling.

### 2.2. Bacterial Strains and Growth Conditions

The bacterial strains used in this study are American type culture collection which is listed in Table 1. All the ATCC* Nocardia* bacterial strains were grown on peptone-yeast-calcium (PYCa) agar [13], R2A agar [27], and Tryptone Yeast Glucose Agar (TYGA) [28]. Three growth media were used to comparatively see the best growth of* Nocardia* species. The PYCa agar was found to be the best media for supporting the growth of* Nocardia* species on the basis of plate count assay. Henceforth, PYCa medium was preferred for maintenance of* Nocardia* cultures at 4°C and was routinely maintained by subculturing every two weeks. After incubation, all the bacteria were enriched in the PYCa broth and stored at 4°C as per the method described by Petrovski et al. [13].

### 2.3. Isolation and Purification of Nocardiophage

Nocardiophages were isolated from wastewater sample collected around Nagpur, India. In brief, 20 mL wastewater sample from each different sampling sites was subjected to centrifugation at 3000 ×g for 20 mins. After centrifugation the supernatant was filtered through cellulose acetate membrane filters (0.22 *μ*m) to remove bacterial cells. For enrichment of bacteriophages, 1 mL of each filtered sample was inoculated with the 50 mL of PYCa broth of mixed bacterial culture and incubated at 30°C [13]. After enrichment the bacterial cells were first centrifuged at 5,000 ×g for 10 min, the supernatant was filtrated through 0.22 *μ*m cellulose acetate filter paper. For plaque assay method, 1 mL filtered sample was mixed with 1 mL of host culture of each species and poured onto autoclaved petriplates after mixing the melted PYCa agar [29, 30]. Plates were then incubated at 30°C for 2 days as per the method of Petrovski et al. [13].

### 2.4. Nocardiophage DNA Isolation

DNA of all the phages NOC1, NOC2, and NOC3 were isolated using SDS-proteinase K method as described by Petrovski et al. [13].

### 2.5. Phage Host Range Determination

All three* Nocardia* species were subjected to host range determination by challenging against nocardiophages as per the method described by Petrovski et al. [13]. Phage recovery and purification was achieved with their respective host, namely,* N. rhodochrous, N. amarae,* and* N. pinensis,* as described by Petrovski et al. [13]. The phage characterization was achieved by nucleotide sequencing and analysis.

### 2.6. Phage Nucleotide Sequence Analysis

Nocardiophage DNA was genome sequenced using the Roche GS FLX genome sequencer and titanium chemistry by Genoseq (CA). The pyrosequencing reads were assembled using the gsAssembler (Roche Applied Science, Indianapolis, IN). All resulting single contigs obtained for each phage had a minimum of 50 times read coverage. After getting the genome sequences, the nucleotide sequences were subjected to analysis like NCBI-BLAST and Open Reading Frame (ORF) prediction using NCBI's ORF Finder tool available at http://www.ncbi.nlm.nih.gov/gorf/gorf.html. The G+C (%) content for the sequences was evaluated using BitGene genetic analysis software available at http://bitgene.com/gene-analysis.shtml.

### 2.7. Nucleotide Sequence Accession Number

The partial genome sequences for* Nocardia* bacteriophages NOC1, NOC2, and NOC3 have been deposited in GenBank under accession numbers KF879861, KF879862, and KF879863, respectively.

## 3. Results and Discussion

### 3.1. Isolation and Purification of Nocardiophages

**Figure 1:**
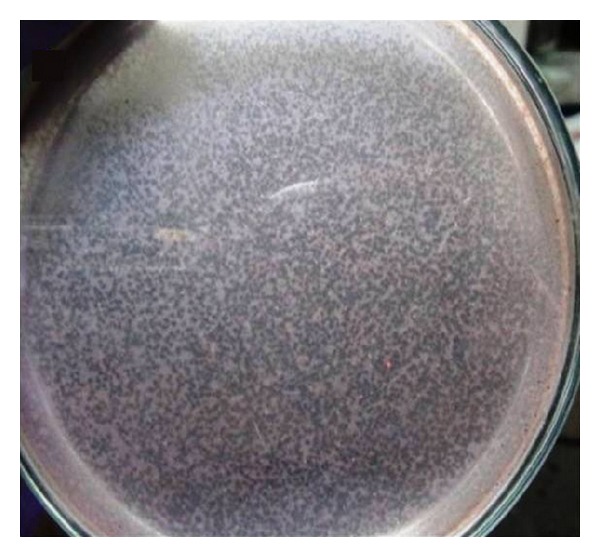
Plaque assay of phage; isolated nocardiophage showing clear plaques on ATCC* Nocardia bacteria.*

Petrovski's method was followed for the isolation and purification of bacteriophages. Single plaques were observed after 2 days incubation at 30°C. The quantitative results of plaque assay are summarized in Table 2 indicating phage growth. A representative culture plate of plaque assay is presented, wherein the plaques were picked and subjected to further isolation and purification of bacteriophages (Figure 1). On the basis of growth of bacteriophages on* N. rhodochrous*,* N*.* amarae*, and* N. pinensis* (reclassified as* Skermania piniformis*) phages were named as NOC1, NOC2, and NOC3, respectively. Further, plaques were purified by four rounds of dilution and reisolation to ensure that each plaque resulted from a single type of bacteriophage. The plates which were showing the excellent visual growth were taken for the purification of bacteriophages.

### 3.2. Phage Host Range Determination

Phage host range was determined by challenging each of the three* Nocardia* species against individual phage on PYCa media. The determination of host range of bacteriophages against nocardioforms is summarized in Table 3. Briefly, NOC2 (isolated from ETP, Nagpur) was able to grow on the two species of* Nocardia,* namely,* N. amarae* and* N. pinensis*; notably few plaques of NOC2 were seen against the* N. pinensis*. NOC1 and NOC3 were able to propagate only on their host, that is,* N. rhodochrous* and* N. pinensis (Skermania piniformis)*, respectively. NOC2 had relatively broader host range when compared to NOC1 and NOC3. Although NOC3 was able to show slight growth on* N. rhodochrous* but clear lytic plaques were not observed, the NOC3 phage did induce a turbid lysis on* N. rhodochrous* so it was not consider as NOC3's host.

### 3.3. Phage DNA Sequence Analysis

The nucleotide sequencing of* Nocardia* bacteriophages NOC1, NOC2, and NOC3 resulted in generating the partial sequence of 34189 bp, 28456 bp, and 28465 bp, respectively. The nucleotide sequences of* Nocardia* bacteriophages NOC1, NOC2, NOC3 have been submitted to GenBank under accession number [GenBank:KF879861], [GenBank:KF879862], and [GenBank:KF879863], respectively. The results of nucleotide sequence analysis done using various bioinformatics tools like NCBI-BLAST, NCBI's ORF Finder, and BitGene genetic analysis software are summarized in Table 4. The table summarizes the G+C content in mol%, number of ORFs over 100 bp and percent identity for bacteriophages NOC1, NOC2, and NOC3 with other Genbank submissions.

The major aim of this study was to isolate and characterize the nocardiophages which may reduce the foaming problem in WWTP in India. In the present research bacteriophages NOC1 and NOC3 were isolated from ETP of dairy industry and NOC2 phage was isolated from ETP, Nagpur, India. At the DNA level, NOC1 [GenBank:KF879861] was found to have 99% identity with* Gordonia* phage GTE7 [GenBank:JN035618] which was reported to reduce the stable foam formation by mycolata strains* G. terrae, Gordonia amictia* (Ben607), and* Nocardia asteroides* (Nast23) under laboratory conditions [15]. NOC2 [GenBank:KF879862] was found to have 99% identity with Gordonia phage GTE2 [GenBank:HQ403646] which was reported as lytic for* Gordonia terrae, Rhodococcus globerulus, Rhodococcus erythropolis, Nocardia otitidiscaviarum, *and* Nocardia brasiliensis *[14]. NOC3 [GenBank: KF879862] was found to have 99% identity with* Nocardia* phage NBR1 [GenBank:JN116828] which was reported to have* N. otitidiscaviarum *and* N. brasiliensis* as its host [31]. The isolated and characterized phages in this study were showing similarity to those phages reported earlier as active against the stable foam forming bacteria under laboratory conditions. This observation implies that the isolated bacteriophages, namely, NOC1, NOC2, and NOC3, may be helpful in reducing the stable foaming problem in WWTP.

## 4. Conclusion

In the present study, isolates (NOC1, NOC2, and NOC3) bacteriophages were identified and characterized. All three phages had the ability to inhibit the growth of filamentous bacteria. The host range determination showed that NOC1and NOC3 were inhibitory against their respective host while NOC2 phage has slightly broader host range. Further, characterization and analysis showed that these phages showed similarity and identity with the previously reported phages against filamentous bacteria. Thus, the result obtained suggests that the isolated bacteriophages are potent inhibitor of foam forming bacteria. Isolated bacteriophages could be used potentially as a biocontrol agent for foaming problems in activated sludge treatment plants. Potential application of bacteriophage need much attention for the control of foaming as it is the ecofriendly and cost effective approach. Further work is in progress for isolation and characterization of other phages against other foam forming filamentous bacteria.

## Figures and Tables

**Table 1:** Bacterial strains used for the isolation of bacteriophage.

Bacterial strains	Deposited as	ATCC number
*Nocardia rhodochrous *	*Nocardia rhodochrous *	33278
*Nocardia pinensis* (*Skermania piniformis*)	*Nocardia pinensis* Blackall et al.	49497
*Gordonia amarae *	*Nocardia amarae Lechevalier and Lechevalier *	27808

**Table 2:** Isolation of bacteriophages against nocardioforms form various sampling point using plaque assay method.

Sample sites	Different points of sampling	*Nocardia rhodochrous *	*Nocardia amarae *	*Nocardia pinensis* (*Skermania piniformis*)
ETP	AT	−	+^b^	−
	AS	−	+++^b^	−
	SC	−	−	−

Dairy industry	AT	−	−	+++^c^
	AS	+++^a^	−	−
	SC	−	−	+^c^

AT: aeration tank, AS: activated sludge, SC: secondary clarifier, − no visual growth; + appearance of visual growth; +++ excellent visual growth.

^a^NOC1.

^b^NOC2.

^c^NOC3.

**Table 3:** Determination of host range of isolated bacteriophages against nocardioforms.

Bacterial species	Bacteriophage		
	NOC1	NOC2	NOC3
*Nocardia rhodochrous *	+++	−	+/−
*Nocardia amarae *	−	+++	−
*Nocardia pinensis* (*Skermania piniformis*)	−	++	+++

− No visual growth; ++ good visual growth; +++ excellent visual growth.

+/− Slight growth.

**Table 4:** Result of nucleotide sequence analysis for isolated bacteriophages NOC1, NOC2, and NOC3 partial genome.

Isolated bacteriophage partial genome sequences (GenBank accession number)	Length of sequence (bp)	Total G+C content (mol%)	Number of ORF over 100 bp	Identity with other bacteriophage genome sequences (GenBank accession number)	Query coverage (%)	Identity (%)
Nocardia phage NOC1 (KF879861)	34189	56.70	182	Gordonia phage GTE7 (JN035618)	100%	99%
Nocardia phage NOC2 (KF879862)	28456	61.17	150	Gordonia phage GTE2 (HQ403646)	100%	99%
Nocardia phage NOC3 (KF879863)	28465	67.49	147	Nocardia phage NBR1, complete genome (JN116828)	100%	99%
